# Effects of *Azadirachta indica* on neuropathic pain induced by chronic constriction injury to sciatic nerve of Wistar rat

**DOI:** 10.5455/javar.2022.i603

**Published:** 2022-09-29

**Authors:** Parijat Biswas, Monira Shahnaz, Masuma Akhter, Arifa Parvin Ripa, Taskina Ali, Kazi Rafiq

**Affiliations:** 1 Department of Physiology, Ashiyan Medical College Hospital, Dhaka, Bangladesh; 2 Department of Physiology, Bangabandhu Sheikh Mujib Medical University, Dhaka, Bangladesh; 3 Durgapur Health Complex, Rajshahi, Bangladesh; 4 Department of Pharmacology, Bangladesh Agricultural University, Mymensingh, Bangladesh

**Keywords:** *Azadirachta indica*, CCI, neuropathic pain, walking track analysis, cold tail immersion test, von frey test, hot plate test

## Abstract

**Objective:**

The research was designed to assess the consequences of *Azadirachta indica* aqueous leaf extract (AILE) *on* neuropathic pain in Wister rats and the role of the ATP-dependent potassium channel (K_ATP_) as an underlying mechanism.

**Materials and Methods:**

This experimental layout was conducted on Wistar rats (*n* = 120) having 150 to 200 gm of body weight. On the foundation of the experimental design, rats were divided into group I (normal saline, 5 ml/kg/body weight) and group II (sham surgery and treatment with NS), group III [chronic constriction injury (CCI) in the sciatic nerve; and treated with NS], group IV (CCI and treated with AILE 400 mg/kg body weight), Group V (CCI, pretreated with Glibenclamide 15 mg/kg followed by treated with AILE 400 mg/kg). All the treatments were given once daily for a consecutive 21 days via the oral route, except Glibenclamide. Glibenclamide was given once through the intraperitoneal route on the day of the experiment.

**Results:**

Based on the neuropathic pain evaluation test, all groups were again sub-divided into subgroup “a” (walking tract analysis), “b” (cold tail immersion test), “c” (Von Frey test), and “d” (hot plate test). AILE showed a significantly higher sciatic functional index (*p* < 0.05) in walking track analysis, tail flick latency (*p* ≤ 0.05) in the cold tail immersion test, and paw withdrawal threshold (*p* ≤ 0.05) in the Von Frey test compared to CCI control. In addition, a nonsignificant difference in all these above-mentioned variables between the rats with CCI plus AILE and the CCI plus AILE plus glibenclamide group indicated that the K_ATP_ channel was not involved in the beneficial analgesic effects of AILE.

**Conclusions:**

The outcome of the present study indicates that AILE prevented worsening of neuropathic pain after chronic constriction injury in the sciatic nerve of Wistar rats in which the K_ATP_ channel was not involved.

## Introduction

Neuropathic pain exerts a strong as well as a negative influence on both the survival and condition of the patient’s life. Neuropathic pain arises from any defect of the nervous system, which includes both peripheral and central sensitization activities [1,2]. Different clinical conditions such as diabetes, cancer, acquired immunodeficiency syndrome, leprosy, cervical disc protrusion, multiple sclerosis, Guillain Barre syndrome, inflammatory demyelinating polyneuropathies, mononeuritis, damage to the vasa nervorum, deficiency of B vitamins (B_1_, B_6_, and B12), and foraminotomy may cause peripheral neuropathy [3–8]. However, many mechanisms have been proposed as the cause of this neuropathy, such as the involvement of voltage-gated calcium channels [2], voltage-gated sodium channels [9], inflammatory cytokines, receptor signaling, intracellular Ca^2 + ^ion, glial cell activation, ectopic discharge in lesioned fibers, etc. [2]. In addition, the involvement of ATP-sensitive potassium channels (K_ATP_) in peripheral neuropathy has also been reported [10]. This K_ATP_ channel is mainly involved in the perception of pain in neuropathy [11]. They are present in almost every pain pathway [11,12].

Therefore, blocking of these K_ATP_ channels might cause hyperalgesia by continuing depolarization of the cell membrane of the pain pathway [10–12]. That is why Glibenclamide (a known K_ATP_ channel blocker) has been used to explore the mechanism of analgesia, observed with different vitamins B [13] as well as different antidepressants [11]. Various animal models have been created to better understand the mechanisms behind the development and maintenance of this neuropathy [14]. These animal models have been established to trigger different clinical and pathological circumstances of peripheral neuropathy, among which sciatic nerve ligation or chronic constrictive injury (CCI) to the sciatic nerve is the most important. This animal model resembles the features of neuropathic exertion in humans [15]. For alleviation of this agonizing neuropathic pain, different drugs, such as tricyclic antidepressants, anticonvulsants, as well as opioids, are the present treatment regimens [16]. However, a wide range of unwanted effects is related to the regular use of these above-mentioned drugs, which reduce their effectiveness in the management of neuropathy [17].

To minimize the unwanted effects of conventional medicines on neuropathy-related pain, nowadays, different medicinal herbs are highlighted due to their wide range of beneficial applications with fewer side effects. Within them, *Azadirachta indica* (AI) is familiar for its broad range of biologic activities. AI leaf extract has anti-inflammatory [18–20], antiviral [21], hepatoprotective [22], anticancer [23,24], antioxidant [25], antifungal [26], antidiabetic [27], and neuroprotective [28] properties. these above-mentioned studies have been done with different dosages and durations of *A. indica* aqueous leaf extract (AILE) on different activities, but most recently, Kandhare et al. [28] exhibited statistically significant neuroprotection with 400 mg/kg of AILE for a consecutive 21-day administration in the CCI model. In addition, no toxic effect was found in mice treated with AI, even up to 2,000 mg/kg oral administration [29].

Many studies have been done on the different actions of AILE, but only two studies have been found to evaluate the neuroprotective action of this medicinal herb. According to the information we have, no published data was available to explore the involvement of K_ATP_ channels as an underlying neuroprotective mechanism of AILE. Therefore, the current study has been organized to assess the effect of AILE (400 mg/kg for 21 days) on neuropathy in the Wister rat and also to assess whether ATP-dependent potassium channels (K_ATP_) are involved as an underlying mechanism.

## Materials and Methods

### Duration and year of study

Between March 2018 and February 2019, the study was conducted in conjunction with the Departments of Physiology at Bangabandhu Sheikh Mujib Medical University (BSMMU), Dhaka, and the Department of Pharmacology at Bangladesh Agricultural University, Mymensingh. The Institutional Review Board (IRB) of BSMMU approved the study protocol (IRB registration Number: 2560).

### Obtainment and upkeeping of animals

A total of 120 rats weighing between 150 and 250 gm were taken from the Central Animal House, BSMMU. All of the rats were housed in specially constructed plastic cages with three to four rats per cage under a 12/12-h light/dark cycle at the Animal Laboratory of the Department of Physiology at BSMMU [30]. The ambient room temperature was kept between 27°C and 28°C, which is rodent thermoneutral [31]. All the rats had free access to the standard laboratory food [32] cooled boiled water *ad libitum* during acclimatization. To avoid circadian influences [33], all the experimental procedures were performed during the daytime between 08:00 and 16:00 h.

### Animal grouping and dose schedule

**Figure 1.**
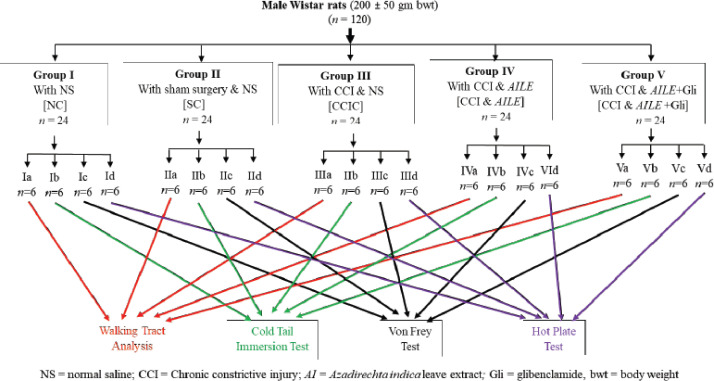
Graphical representation of the experimental design.

Based on treatments, all the rats were randomly divided into the following groups: Group I: normal control consists of 24 rats who were given normal saline (NS, 5 ml/kg) for 21 days in a row. Group II: Sham control [consists of 24 rats; sham surgery was done (open and close, no sciatic nerve injury) and treated with normal saline (NS, 5 ml/kg) for a consecutive 21 days]. Group III: CCI Control (CCIC consists of 24 rats; CCI to the left sciatic nerve was done, followed by NS treatment for a consecutive 21 days). Group IV: CCI *&* AILE (consists of 24 rats. CCI to the left sciatic nerve was done, followed by treatment with AILE(400 mg/kg, orally) for a further 21 days). Group V: CCI & AILE + Gli, (CCI & AILE + Gli; consists of 24 rats). CCI to the left sciatic nerve was done, followed by treatment with AILE(400 mg/kg) for a consecutive 21 days and Glibenclamide (Gli, 15 mg/kg) 10 min before the experiment. Glibenclamide was given once through an intraperitoneal route on the day of the experiment. Based on the neuropathic pain evaluation test, all the groups were further subdivided into subgroups “a”,“b”, “c”, and “d” (*n* = 6 rats in each subgroup) described as: a; Walking tract analysis, b; Cold tail immersion test, c; Von Frey test, and d; Hot plate test. In line with our previous study [34], a graphical representation of the experimental layout is shown in Figure 1.

### Chronic constrictive injury

CCI of the sciatic nerve was done by a previously described procedure [35,36]. At first, anesthesia was done by using ketamine (80 mg/kg) and xylazine (10 mg/kg) mixture which was applied through intraperitoneal injection followed by shaving of the left hind leg region. Then, the animal was placed on a mat at 37°C, where the temperature can be regulated. Alcohol (70%) and iodine solution were applied alternatively to the shaved region to ensure sterilization. The rats were laid down on their chests. Then positioning of the left hind leg was done by elevation and using masking tape on the foot. An incision was made in the skin, 3 to 4 mm below the femoral head. Muscles adjacent to the incision were freed from the skin. The connective tissue was cut in the middle of the gluteus superficialis and biceps femoris muscles by using blunt scissors. The gap was widened between those two muscles, which allows a thorough image of the sciatic nerve. The sciatic nerve (proximal to the sciatic trifurcation) was smoothly released from the surrounding connective tissue by using blunt-tipped forceps and micro-scissors. The sciatic nerve was tied proximal to the trifurcation with three ligatures (chromic gut 4.0). The ligatures were 1 mm apart from each other and were not too tight in nature. Constriction of the nerve was minimal to prevent the arrest of the epineural blood flow. Sutures and staplers (mersilk 5.0) were used to enclose the muscle and fix the skin, respectively. Sterilization of the wound was done by iodine solution and the rat was carefully watched throughout the anesthesia regaining interval by keeping it in an isolated cage with uniform paper sheets (not the typical animal bedding) to prevent animal choking during the anesthetic interval.

### Walking track analysis

The walking track analysis test method was carried out as per previously published methodology [37,38]. Six rats from every group were separated for walking track analysis. Each rat was trained to walk freely on a smooth, flat surface covered by a sheet of ink-absorbing paper and guarded by a wooden plate for 7 days in a row. This test was done every 4 days at interval after CCI induction. On the day of the experiment, just 1 h after the treatment, each rat was kept individually in the above-mentioned corridor on a sheet of ink-absorbing paper after dipping its hind paws in blue ink. Each time, 3 to 4-foot prints of each foot were marked and a measuring scale was used to measure as mentioned below. Then sciatic functional index (SFI) was calculated as follows:

SFI = [-38.3×(EPL-NPL)/NPL] + [109.5×(ETS-NTs)/NTS] + [13.3×(EIT-NIT)/NIT] -8.8 Where NPL = Normal print length; EPL = Experimental print length; NTS = Normal toe spread; ETS = Experimental toe spread; NIT = Normal intermediate toe spread; and EIT = Experimental intermediate toe spread.

### Cold tail immersion test

The cold tail immersion test method was carried out as per previously published methodology [39]. Six rats from each group were separated for the cold tail immersion test. During instrumental acclimatization, each rat was placed in a mechanical restrainer made of plexiglass for 10 min each day for a consecutive 7 days. Then, on the first day of the experiment, at 8.00 a.m., each rat was kept separately in the plexiglass mechanical restrainer for 5 min to adjust to the cage environment. Then the distal 5 cm of the freely hanging tail of the rat was dipped into cold water maintained at 10°C, and the latency period of the tail withdrawal (tail-flick) was documented. The mean of the measurements was gotten from three similar successive operations, conducted at 5 min intervals, and was recorded as the baseline latency, 1 h after the last dose of the treatment (NS/AI). Then again, another tail immersion test was conducted. Here, the mean of three tail withdrawal latencies at a 5 min gap were noted as test latency (TL). To minimize tissue injury, a maximum latency of 15 sec was accounted for as the cut-off time. The antinociceptive effect was expressed as % of the maximum possible effect (% MPE) as follows: [(TL-BL) / (Cut off time-BL)] × 100.

### Von Frey test

The Von Frey test method was carried out as per previously published methodology [24,40,41–45]. Six rats from each group were separated for the Von Frey test. These rats were further acclimatized on a gauge wire mesh surface for 1 h daily for seven successive days.

This test was performed every three days following CCI induction. On the day of the experiment, just 1 h after the treatment, each rat was placed individually on the wide gauge wire mesh surface. Then the calibrated Von Frey filaments (VFF) were touched on the planter surface of the hind paws (both) of the rat in between the first and second metatarsals, approximately 1 cm proximal to the ankle joint. Each VFF (of varying tensile strengths of 2 to 18 gm) was applied three times at a 30-sec gap, and the number of hind paw withdrawals was documented. The next larger VFF was applied unless paw withdrawal occurred in at least two of the three. If the rat fails to withdraw its paw at a maximum force of 18 gm, then no more VFF was applied to prevent tissue destruction.

### Hot plate test

The hot splate procedure was performed as per the previously mentioned method [46]. Six rats from each group were separated for the hot plate test. Along with laboratory acclimatization, these rats were acclimated to the instrument for 1 h daily for seven days experimental days; the rat was left undisturbed for at least 10 min in the instrument box before the test. The first hot plate was heated at a temperature of 54 ± 0.5ºC, and the rats were placed on the heated surface. A cut-off period of 20 sec was taken to avoid any injury to the paw. Then the stop clock was used to check the nociceptive latency, as defined by paw licking or trying to jump out of the glass box. The stop clock began as the rat placed it on the hot plate and continued until the first paw licked or tried to jump out. The analgesia is expressed as Percentage Maximal Possible Effect (%MPE)


$$\%\;\mathrm{MPE}\;=\;\frac{\mathrm{Testlatency}\;–\;\mathrm{Basallatency}}{\mathrm{Cutofftime}\;–\;\mathrm{Basallatency}}\;\times \;100$$

### Data presentation and statistical analysis

Results were demonstrated as mean ± SEM. Statistical evaluation was done by using SPSS (version 23.0). Statistical tests were carried out by analysis of variance, followed by a Bonferroni *posthoc* test. In interpreting the results, *p* ≤ 0.05 was considered to be statistically significant.

## Results

### Sciatic functional index in walking track analysis

**Figure 2.**
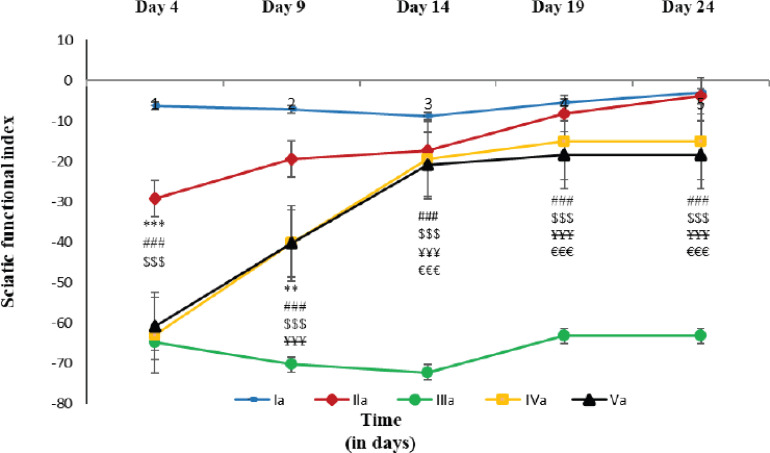
Sciatic functional index in different days of walking track analysis in different groups of rats. Each line symbolizes the mean ± SME for six rats. Ia = rats with normal oral saline (5 ml/kg) for a consecutive 21 days. IIa = treated with open and close surgery followed by oral normal saline (5 ml/kg), IIIa = rats treated with CCI to sciatic nerve followed by oral normal saline (5 ml/kg) for consecutive 21 days, IVa = rats treated with CCI followed by oral AILE (400 mg/kg orally) for consecutive 21 days. Va = rats treated with CCI followed by oral AILE (400 mg/kg) for consecutive 21 days and also with a single dose of intraperitoneal Glibenclamide (15 mg/kg)10 min before AILE on the day of the experiment. * = Ia vs IIa, # = Ia vs IIIa, $ = IIa vs IIIa, ¥ = IIIa vs IVa, € = IIIa vs Va, and £ = IVa vs Va.

Here, the SFI in walking track analysis was used to determine the sensorimotor impairment of the sciatic nerve. The SFI in the AI experimental group was significantly lower (*p* ≤ 0.001) in comparison to those of the CCI control group on days 9, 14, 19, and 24. Moreover, there was no significant difference in mean values between the AI experimental group and the Glibenclamide experimental group on any day of the experiment (Fig. 2).

### Tail flick latency of tail immersion test

**Figure 3.**
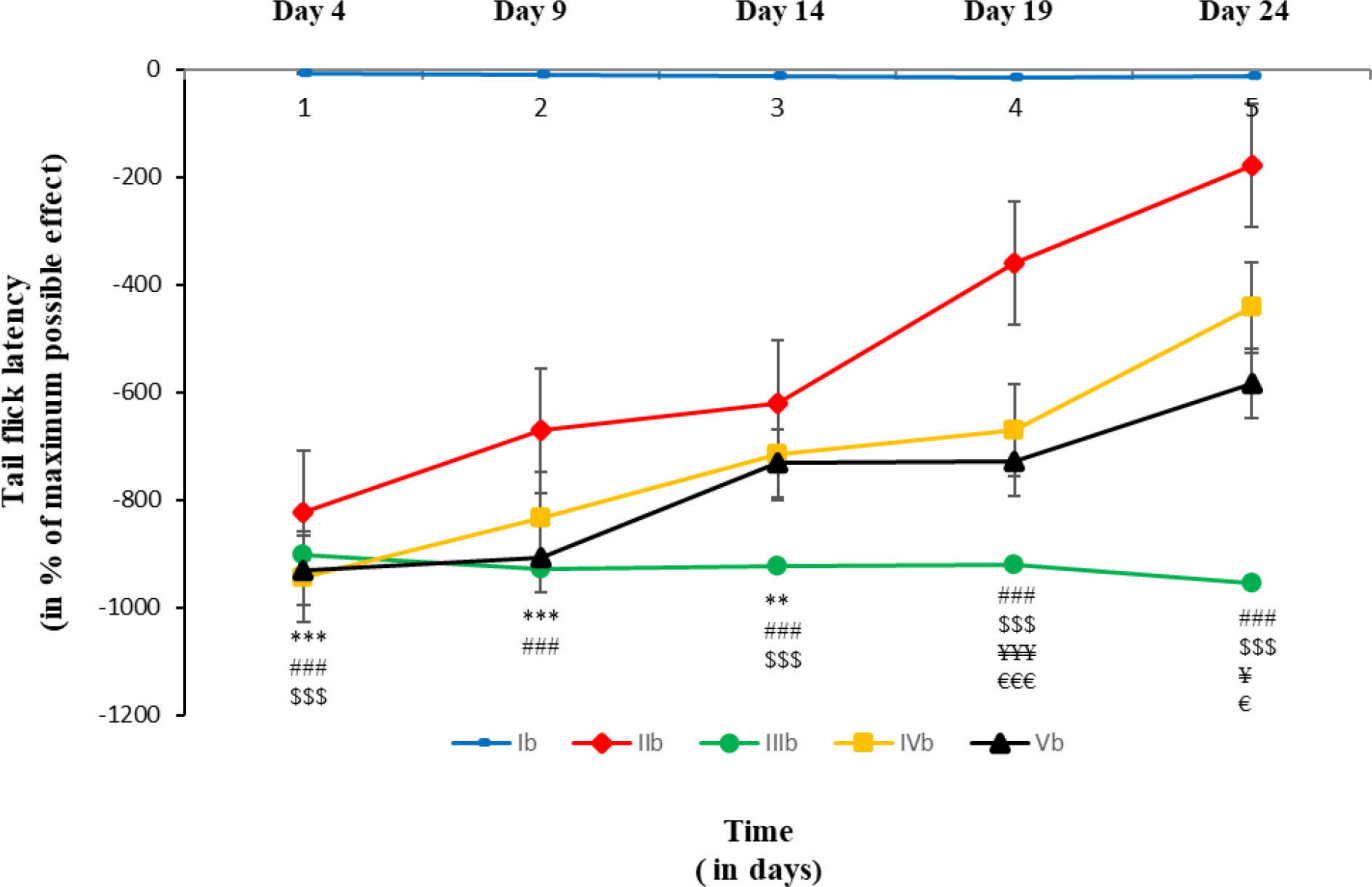
Tail flick latency on different days of tail immersion test in a different group of rats. Each line symbolizes mean ± SEM for six rats. Ib = rats with oral normal saline (5 ml/kg) for consecutive 21 days. IIb = rats with open and close surgery followed by normal oral saline (5 ml/kg) for consecutive 21 days. IIIb = rats with CCI to sciatic nerve followed by oral normal saline (5 ml/kg) for consecutive 21 days. IVb = rats with CCI followed by oral AILE (400 mg/kg) for consecutive 21 days. Vb = rats with CCI followed by oral AILE (400 mg/kg) for consecutive 21 days and also with a single dose of intraperitoneal Glibenclamide (15 mg/kg) 10 min before AILE on the day of the experiment. * = Ib vs IIb, # = Ib vs IIIb, $ = IIb vs IIIb, ¥ = IIIb vs IVb, € = IIIb vs Vb, and £ = IVb vs Vb.

We evaluated the effect of AI on neuropathic pain by tail withdrawal latency in a tail immersion test. As shown in Figure 1, here, the tail withdrawal latency in the AI experimental group was significantly lower in comparison to those of the CCI control group on day 19 (p ≤ 0.05) and day 24 (p ≤ 0.001). Moreover, there was no significant difference in mean value between the AI experimental group and the glibenclamide experimental group (Fig. 3).

### Paw withdrawal threshold in Von Frey Test

**Figure 4.**
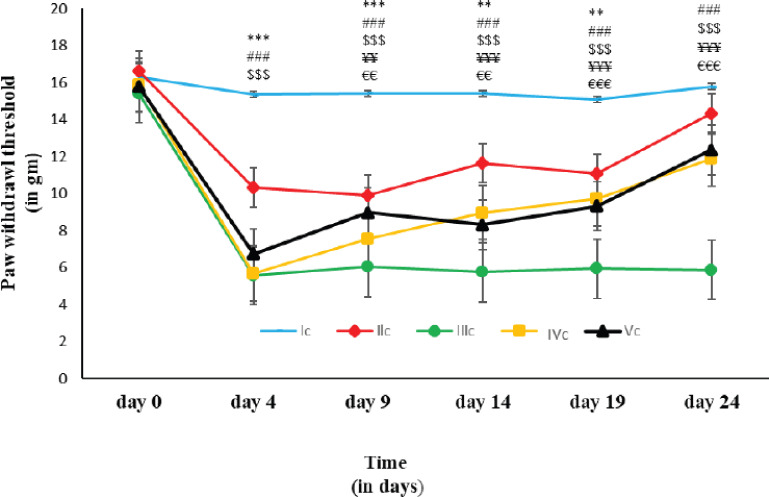
Paw withdrawal threshold on different days of the von Frey test in different groups of rats Each line symbolizes the mean ± SEM for six rats. Ic = rats with oral normal saline (5 ml/kg) for a consecutive 21 days. IIc = rats with open and closed surgery followed by oral normal saline (5 ml/kg) for a consecutive 21 days. IIIc = rats with CCI to the Sciatic Nerve followed by oral normal saline (5 ml/kg) for a consecutive 21 days. IVc = rats with CCI followed by oral AILE (400 mg/kg) for a consecutive 21 days. Vc = rats with CCI followed by oral AILE (400 mg/kg) for consecutive 21 days and also with single dose of intraperitoneal Glibenclamide (15 mg/kg)10 min before AILE on the day of the experiment. * = Ic vs IIc, # = Ic vs IIIc, $ = IIc vs IIIc, ¥ = IIIc vs IVc, € = IIIc vs Vc, and £ = IVc vs Vc.

The effect of oral administration of AI on neuropathic pain and the effect of Glibenclamide over the AI group in the Von Frey test by paw withdrawal threshold. The result showed that the paw withdrawal threshold of the AI experimental group was significantly lower in comparison to those of the CCI control group on day 9 (*p* ≤0.01), 14 (*p* ≤ 0.001), 19 (*p* ≤ 0.001), and 24 (*p* ≤ 0.001). Moreover, there was no significant difference in mean values between the AI experimental group and the Glibenclamide experimental group on any day of the experiment (Fig. 4).

### Reaction time in hot plate test

**Figure 5.**
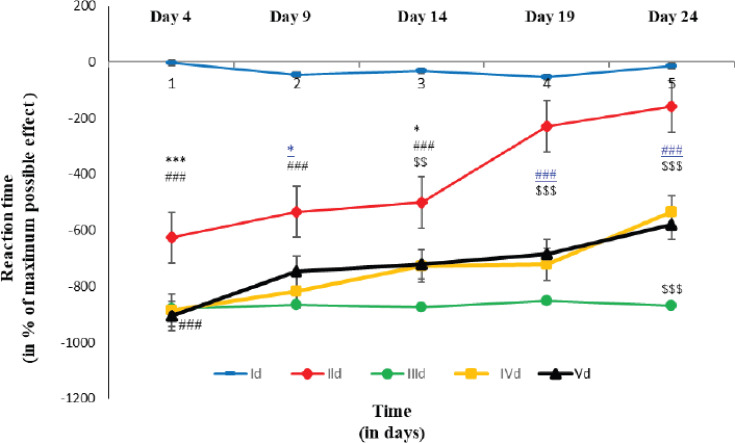
Reaction time on different days of hot plate tests in different groups of rats each line symbolizes the mean ± SEM for six rats. Id = rats with oral normal saline (5 ml/kg) for a consecutive 21 days. IId = rats with open and closed surgery followed by oral normal saline (5 ml/kg) for a consecutive 21 days. IIId = rats with CCI to the sciatic nerve followed by oral normal saline (5 ml/kg) for consecutive 21 days. IVd = rats with CCI followed by oral AILE (400 mg/kg) for a consecutive 21 days. Vd = rats given CCI followed by oral AILE (400 mg/kg) for 21 days in a row, as well as a single intraperitoneal Glibenclamide (15 mg/kg) dose 10 min before AILE on the day of the experiment. * = Id versus IId, # = Id versus IIId, $ = IId versus IIId, ¥ = IIId versus IVd, € = IIId versus Vd and £ = IVd versus Vd.

Here, the thermal allodynia was assessed by the latency of reaction time in the hot plate test. The reaction times in the AI experimental groups were not significantly lower in comparison to those of group CCI control on any day of the experiment. Moreover, there was no significant difference in mean values between the group AI experimental and group glibenclamide experimentation on any day of the experiment (Fig. 5).

## Discussion

The present experimentation has been undertaken to assess the effects of AILE on neuropathy and on K_ATP_ channels. For this, 120 Wistar rats were studied to observe this medicinal herb’s effects on sensorimotor dysfunction of the sciatic nerve (SFI), cold allodynia, mechanical allodynia, and heat hyperalgesia.

In the present study, loose ligation around the sciatic nerve might compress the nerve fiber, causing neural damage [46]. Based on previous reports the damaged cell released various inflammatory mediators including prostaglandin (PG) E2 [47–50] augmenting chemotaxis of inflammatory cells (macrophage, microglia, monocyte, helper T cell) [51]. They release inflammatory mediators including interleukine-1β (IL-1β), tumor necrosis factor-α (TNF-α), interleukin 6 (IL-6), bradykinin, substance P, nerve growth factor, PG [9,47,50]. These mediators bind with their specific receptors on the peripheral end of nociceptors followed by activation of the signaling pathway, p38 mitogen-activated protein kinase (MAPK), resulting in the insertion of Na^ + ^channels on the nociceptors’ membrane [9,52]. Along with p38 MAPK, TNF-α also activate nuclear factor kappa B (NF-κB), causes activation of genes related to cell death [B-cell lymphoma 2 (BCL-2) B-cell lymphoma extra-large (BCL-XL)] [48,50,53]. In addition to the accumulation of inflammatory mediators causes an increment in concentration of extracellular H + ion to lower the pH of ECF [47]. This acidic pH activates transient receptor vanilloid 1 by proton sensor, causing further insertion of Na^ + ^channels in the injured peripheral nerve terminal [47]. All these mechanisms ultimately cause a decrement of the activation threshold and an increment of excitability of these peripheral nerves, causing enhancement of Ca^2 + ^channel trafficking in the central terminal of the nociceptor. The activated central terminal of the nociceptor releases glutamate, ATP, and other mediators causing prolonged post synaptic depolarization of dorsal root ganglia (DRG) [9]. Besides this, discharge from the primary injured nerve causes an increment of mitochondrial superoxide (O·¯_2_) production followed by an accumulation of toxic hydroxyl radical (·OH). These increased toxic Reactive Oxygen Species, might cause membrane damage, glial activation, and central sensitization [54]. On the other hand, glutamate (released from the central terminal of nociceptors) causes relief of physiological Mg^2 + ^block of N methyl D-aspartate receptors, followed by raised excitability of DRG with the subsequent intensification of pain transmission [9]. Along with glutamate, the central terminal of the nociceptor also releases ATP, which binds to purinoceptors (P2X4) on microglia and activates them, also leading to p38 MAPK activation followed by prolonged postsynaptic depolarization of DRG [9]. Activated macrophage engulf both injured and uninjured axons and induce further immunoreaction [9,49]. In addition, retrograde transport of TNFα in DRG increases the membrane conductance of K + ion in a non-voltage gated manner and causes overall hyperexcitability [55]. In our experimental rats with CCI-induced neuropathic pain, all or any of the aforementioned mechanisms may contribute to an increase in pain transmission along with sensorimotor dysfunction of the sciatic nerve, cold allodynia, mechanical allodynia, and heat hyperalgesia.

It has been reported that AILE had a significant anti-inflammatory effect by reducing 5-HT and PGE1 in carrageenan-induced hind paw inflammation [56] and by reducing different neural inflammatory mediators (TNFα, IL-1β) in a partial sciatic nerve ligation evoked neuropathic pain model [28]. This extract also showed significant antioxidation activities by reducing lipid peroxidation [28,57,58], increasing superoxide dismutase level [28,58,59], elevating the reduced glutathione (GSH) [28,58,59], as well as by increasing catalase activity [58] in different animal models of neuropathy and neuropathic pain. This herbal extract also caused significant anti-apoptosis by inhibiting the TNFα-triggered induction of NF-κB [28,59] and by increasing BCL2 mRNA in neuropathic rats [28]. It also inhibited central sensitization of DRG by reducing the neural Ca2 + content in another neuropathic pain model of rats [28]. In addition, suppressing the inflammatory mediators as well as increasing antioxidants, in addition to anti-apoptosis or inhibition of the central sensitization, might be the causes of the prevention of dysfunction of the sciatic nerve, cold allodynia, and mechanical allodynia after AILE administration in our rats with CCI induced neuropathic pain. However, we could not explain the mechanisms of non-attenuation of heat hyperalgesia by AILE in our neuropathic rats. Therefore, for further studies with different doses and durations of AILE, evaluation of serum inflammatory markers and antioxidant activities both before and after administration of AILE is needed to explore the molecular mechanism through which AILE exerts its beneficial analgesic effects in this pathophysiological condition in a rat model.

## Conclusion

The results of the current study elucidate that AILE prevented worsening of neuropathy in rats and that K_ATP_ channels may not be involved in this pathophysiological condition in chronic constriction injury in the sciatic nerve of the Wistar rat model.

## List of abbreviations

ECF, extracellular fluid; GSH, Glutathione CCI, chronic constriction injury; K_ATP,_ ATP-sensitive potassium_;_ AILE, *Azadirachta indica* aqueous leaf extract ; DRG, dorsal root ganglia; VFF, Von Frey filaments; SFI, sciatic functional index; BSMMU, Bangabandhu Sheikh Mujib Medical University; IRB, Institutional Review Board.
